# Cranial morphology in flying squirrels: diet, shape, and size disparity across tropical and temperate biomes

**DOI:** 10.1186/s12983-025-00556-4

**Published:** 2025-03-11

**Authors:** Álvaro Quesada, Manuel Hernández Fernández, Iris Menéndez

**Affiliations:** 1 Departamento de Geodinámica, Estratigrafía y Paleontología, Facultad de Ciencias Geológicas, Universidad Complutense de Madrid, C/ José Antonio Nováis 2, 28040 Madrid, Spain; 2 Departamento de Cambio Medioambiental, Instituto de Geociencias (UCM, CSIC), C/ Severo Ochoa 7, 28040 Madrid, Spain; 3 Museum für Naturkunde , Leibniz Institute for Evolution and Biodiversity Science, Invalidenstrasse 43, 10115 Berlin, Germany

**Keywords:** Flying squirrels, Morphology, Geometric morphometrics, Skull, Diet, Allometry, Biomes

## Abstract

**Background:**

Species richness increases gradually as latitude decreases, however, the explanation for this phenomenon remains unclear. Ecological hypotheses suggest that greater niche diversity in tropical biomes may facilitate the coexistence of a larger number of species. The close relationship between species morphology and ecology can lead to a greater morphological disparity in tropical biomes.

**Methods:**

In this study, we used 2D geometric morphometric techniques on the ventral view of the cranium of flying squirrels (Pteromyini, Sciuridae) to determine the relationship between diet and cranial morphology and to evaluate if morphological disparity is higher in tropical biomes.

**Results:**

The results show that diet has a significant impact on cranial shape and size, with large, wide and robust crania in folivorous and generalist species, while frugivorous species tend towards smaller and narrower crania, and nucivorous have a wide variability. This suggests that biomes with more available dietary niches would show greater morphological disparity. However, we found no statistical differences in shape and size disparity among biomes or between observed and simulated disparity based on species richness.

**Conclusions:**

Our results show that there are not disparity differences between tropical and temperate biomes, even when temperate biomes are less rich than tropical ones, suggesting that the quantity of available niches may not be the key factor in generating morphological disparity. Instead, it could be the presence of extreme niches that demand specialised adaptations for exploitation, which might be of greater significance. A greater importance of size-changing adaptations would decrease shape disparity in biomes with many niches.

**Supplementary Information:**

The online version contains supplementary material available at 10.1186/s12983-025-00556-4.

## Introduction

Biodiversity is not evenly distributed across the planet. Instead, there is a marked latitudinal gradient, with more species richness towards the equator than towards the poles [43, 95, 127]. Furthermore, this trend is recognisable in both hemispheres, in marine and terrestrial species, and in different taxa [60] such as mammals [17, 64], birds [55], insects [40], trees [69] or bacteria [45]. There has been extensive debate on which factors may explain this biogeographic pattern [60, 85]. The multiple proposed hypotheses to explain this phenomenon can be grouped into four types: evolutionary, historical, geographic, and ecological [30, 85]. The evolutionary hypotheses focus on speciation and extinction rates, while historical hypotheses focus on the extension and duration of tropical biomes throughout time. The geographic hypotheses focus on geometric constraints imposed on species ranges, and ecological hypotheses focus on the ecological factors that allow the coexistence of species and the maintenance of their diversity.

One influential ecological hypothesis suggests that tropical habitats offer a greater number of niches for species [75]. This idea proposes that niche packing enables more species to coexist [98, 126], thus increasing the potential for diversification in the tropics. Under this view, we might expect greater ecomorphological differentiation of species living in tropical areas, as species adapt to the exploitation of different resources, a pattern that has been observed in different taxa [24, 61, 70, 99, 109, 115, 120, 125].

Ecomorphological adaptations for the use of different resources can be reflected in bone morphology. For example, vertebrate cranial morphology is greatly related to the food consumed by species [33, 41, 50, 71, 104, 121], which varies in availability along the latitudinal gradient [109, 114]. Additionally, there is a relationship between body size and diet. For example, folivores generally tend to have large body sizes, as plant matter is hard and low in nutrients, this requires longer processing time and the ingestion of large amounts of food [18, 26], which in turn favours having a large gut capacity [27, 35]. Folivores also require more muscular strength for mastication of this hard and abrasive food, which is achieved by having robust masticatory muscles, involving large insertion areas in the jaw and cranium [106]. On the other hand, frugivores are associated with a less robust cranium, since they need less bite force [8]. In addition, some groups usually present small canines and cheek teeth, which contrast with a long incisor tooth row [8, 44], due to the importance of the anterior teeth in these species [44].

A greater diversity of available food resources and niche packing in tropical biomes could therefore generate communities that not only have a greater number of species but also show greater cranial morphology variance than temperate regions. Flying squirrels (Pteromyini, Sciuridae) are a good model to test this hypothesis, as they represent a monophyletic group consisting of 52 species and 14 genera [132] and are present in both tropical and temperate biomes. Most species are distributed in South and Southeast Asia although there are two species endemic to North America (*Glaucomys sabrinus* y *Glaucomys volans*) and another (*Pteromys volans*) which inhabits northern Eurasia [124]. The diet of flying squirrels is highly diverse, for example *Eupetaurus cinereus* feeds only on pine needles [135], *Glaucomys volans* is mainly nucivore [88], *Glaucomys sabrinus* eats lichens and fungi [79], and *Iomys horsfieldii* is mainly frugivore [54, 89]. Therefore, we expect that cranial shape displays differences depending on the species diet, size and the interaction between these two factors.

The main objective of this study was to assess whether the cranial morphological disparity of flying squirrels in tropical biomes, which offer a greater variety of dietary niches, is higher than what would be expected by chance. In order to do that, we first evaluated the relationship between cranial morphology and diet within the Pteromyini tribe. Once we confirmed this relationship, we assessed whether the observed levels of morphological disparity in each biome differed from the expected values based on their flying squirrel richness. For these purposes, we used geometric morphometrics to measure cranial morphology, which is able to transfer most of the morphological information on the studied taxa to quantitative variables [4, 81, 101, 136], and Monte Carlo simulations to generate aleatory models of the expected disparity for each biome [78].

## Materials and methods

### Samples

We photographed crania in ventral view of 151 specimens, belonging to 35 extant species of Pteromyini (67,31% of the total number of species), which represent all the 14 genera of the tribe (Table 1). We chose the ventral view because of the presence of the dental row, whose morphology is closely related to diet, and because of the greater availability of anatomical discrete points convenient for their selection for the geometric morphometric analysis with the adequate repeatability, homology, and shape coverage. Photographs were taken by I.M. to specimens from the American Museum of Natural History (AMNH) and from the National Museum of Natural History (USNM). Data of *Biswamoyopterus laoensis* and *Eupetaurus cinereus* were obtained from images in the literature with the same orientation [80, 105].

**Table 1:** List of species sampled, number of specimens of each species (N), diet, and occupied biomes. Diet categories (Table 2): Folivore 1 (Fol_1.), Folivore 2 (Fol_2.), Frugivore (Frug.), Nucivore (Nuc.), Generalist (Gen.), no information available (No info.). Biomes follow Walter’s [128] classification: evergreen equatorial rainforest (I), tropical deciduous woodland (II), savanna (II/III), temperate evergreen forest (V), broad-leaf deciduous forest (VI), taiga (VIII). Biomes that do not include any flying squirrel species, such as subtropical desert (III), sclerophyllous woodland and shrubland (IV), steppe/cold desert (VII) and tundra (IX), were omitted

Specie	N	Diet	I	II	II/III	V	VI	VIII
*Aeretes melanopterus*	2	No info	–	–	–	–	–	1
*Aeromys tephromelas*	4	Nuc	1	–	–	–	–	–
*Aeromys thomasi*	1	Frug	1	–	–	–	–	–
*Belomys pearsonii*	2	Fol_1	1	1	–	1	1	–
*Biswamoyopterus laoensis*	1	No info	–	1	–	–	–	–
*Eoglaucomys fimbriatus*	8	Nuc	–	–	1	1	–	1
*Eupetaurus cinereus*	1	Fol_2	–	–	–	–	–	1
*Glaucomys sabrinus*	10	Nuc	–	–	–	–	1	1
*Hylopetes alboniger*	7	Frug	1	1	–	1	–	1
*Hylopetes bartelsi*	1	No info	1	–	–	–	–	–
*Hylopetes nigripes*	4	No info	–	1	–	–	–	–
*Hylopetes phayrei*	6	Frug	1	1	–	–	–	–
*Hylopetes platyurus*	7	Gen	1	–	–	–	–	–
*Hylopetes sagitta*	8	No info	1	1	–	–	–	–
*Hylopetes spadiceus*	10	No info	1	1	–	–	–	–
*Iomys horsfieldii*	1	Nuc	1	–	–	–	–	–
*Iomys sipora*	1	No info	1	–	–	–	–	–
*Petaurillus kinlochii*	4	No info	1	–	–	–	–	–
*Petaurista albiventer*	1	Gen	1	1	–	1	–	–
*Petaurista alborufus*	5	Gen	1	1	–	1	1	–
*Petaurista elegans*	3	Gen	1	1	–	1	–	1
*Petaurista lena*	3	Fol_1	1	1	–	1	–	1
*Petaurista leucogenys*	6	Gen	–	–	–	1	1	–
*Petaurista petaurista*	10	Fol_1	1	1	–	1	–	1
*Petaurista philippensis*	5	Fol_1	1	1	1	1	1	–
*Petaurista yunanensis*	5	No info	1	1	–	1	1	1
*Petinomys crinitus*	3	No info	1	1	–	–	–	–
*Petinomys fuscocapillus*	3	Gen	1	1	–	–	–	–
*Petinomys hageni*	2	No info	1	–	–	–	–	–
*Petinomys lugens*	4	No info	1	–	–	–	–	–
*Petinomys setosus*	4	Nuc	1	1	–	–	–	–
*Petinomys vordermanni*	6	Nuc	1	–	–	–	–	–
*Pteromys volans*	6	Gen	–	–	–	1	1	1
*Pteromyscus pulverulentus*	5	Gen	1	–	–	1	–	–
*Trogopterus xanthipes*	2	Fol_2	–	–	–	1	1	1
Total	151		26	17	2	14	8	11

### Diet categorization

The most abundant food components in the flying squirrels’ diet are leaves, fruits, nuts and seeds. Depending on the presence or absence of these foods in the diet, we determined five diet categories (Table 2). Because both leaves and nuts/seeds are more abrasive than fruits, and food abrasiveness probably has a relevant incidence on cranial morphology, we defined folivorous, nucivorous and generalist diets by the presence or absence of these two more demanding food items, although they may also include fruits. Frugivores, on the contrary, were defined by the exclusive consumption of flesh fruits. Information about the diet of each squirrel species (Table 1) was obtained from Koprowski et al. [66] and has been completed with the literature listed in Table S1.

**Table 2:** Description of the diet categories. The categories assigned to the sampled species can be seen in Table 1

Diet categories	Description
Folivore 1	Diet that includes leaves and sprouts but not nuts and hard seeds
Folivore 2	Diet based mainly on very abrasive leaves (e.g. conifer needles) but not nuts and hard seeds
Frugivore	Diet based mainly on flesh fruit but not nuts, seeds or leaves
Nucivore	Diet that includes hard seeds and/or nuts but not leaves
Generalist	Diet that includes leaves, hard seeds and/or nuts
No info	No information available

### Biome occupancy

To determine the biome occupancy of each species, we used the biome classification of Walter [128], shown in Table 1. We used the species distribution collected in Koprowski et al. [66], and the biomes distribution mapped by Allué Andrade [6]. Following Hernández Fernández [57], if 15% or more of the species distribution was within a biome, we considered that that species inhabits that biome. Furthermore, if the species distribution occupied 50% or more of a climatic dominion, it was also considered to occupy the corresponding biome. Following Hernández Fernández [57], a climatic dominion is a continuous terrestrial area within one climate zone only. For instance, the equatorial rainforest biome present in Africa consists of two different climatic dominions, the central area of the Congo River Basin and the western coast of the Gulf of Guinea [37, 128]. Finally, we also considered the sequence of altitudinal vegetation belts in mountain areas, which is analogous to the latitudinal distribution of biomes [86].

### Cranial shape and size

**Fig. 1:**
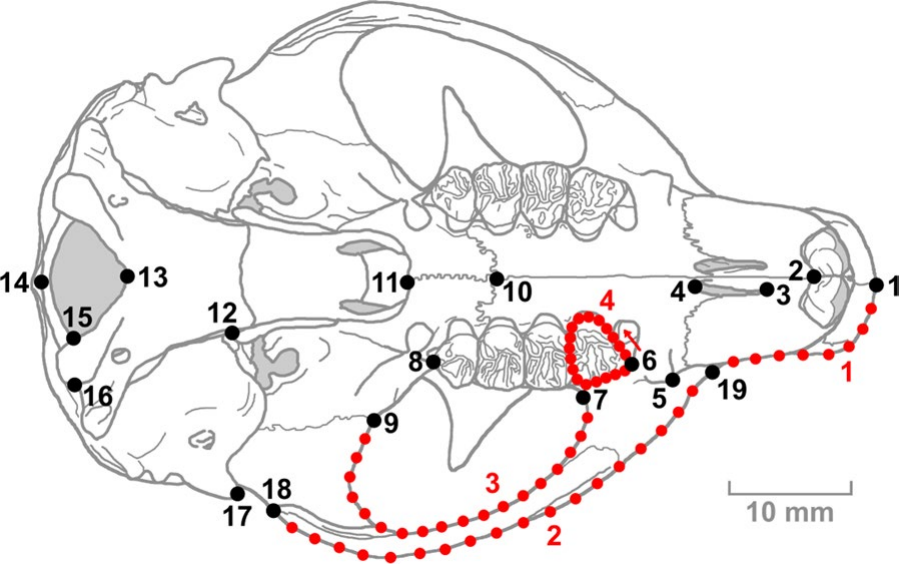
Landmarks (black points) and semilandmarks (red points) on a cranium of *Petaurista lena* in ventral view. See Table 3 for the definition of each of them

The placement of landmarks and semilandmarks was carried out using the *StereoMorph* package in the R environment [91, 97]. We selected 19 landmarks in the cranium and semilandmarks forming four curves (Fig. 1, Table 3) based on previous schemes [19, 20, 74, 93]. To accurately capture the morphology of these curves, we placed 8 semilandmarks for curve 1 and 18 for curves 2, 3 and 4. Curve 1 represents the contour of the premaxilla, which varies in some squirrels genera due to the insertion of the masseter muscles [123], and whose length has been related to the type of diet in other groups of herbivorous mammals [63, 87, 112, 121]. Curves 2 and 3 describe the internal and external curve of the zygomatic arch, where the masseter muscles (deep masseter and zygomaticomandibularis are inserted. These muscles are particularly relevant in mastication in squirrels, along with the superficial masseter, which is inserted in the masseter tubercle [10, 31, 32, 123]. Although the temporalis muscles cross through the zygomatic fossa to insert into the coronoid process, the space they occupy is very small [10, 123] and their role in mastication is mainly the stabilisation of the mandible [32]. Finally, curve 4 constitutes the outline of the fourth upper premolar (P4), which has the best orientation in the ventral view of the cranium, since the occlusal surfaces of the other teeth are slightly oblique. As the cranium is a bilaterally symmetrical structure, landmarks and curves were recorded only on one side of the cranium to avoid redundant information that could introduce error [65], and then we made a specular duplication of landmarks and semilandmarks to create the complete cranial structure. This avoids obtaining differences between species due to differences in the bilateral symmetry of the crania caused by measurement error or individual variation [65].

**Table 3:** Numeration and definition of the landmarks and the curves used in this work (Fig. 1)

Landmark	Definition of the landmark
1	Anterior (mid-sagittal) point of the premaxilla
2	Midpoint of the tooth sockets of the incisors
3	Anterior end of the incisor foramen
4	Posterior end of the incisor foramen
5	Tip of the masseter tubercle
6	Anterior end of the fourth upper premolar
7	Maximum curvature at the internal zygomatic arch
8	Posterior end of tooth row
9	Anterior end of the suture between the alisphenoid and the squamosal zygomatic process
10	Suture between maxilla and palatine in the mid-sagittal plane
11	Posterior end of the suture between right palatine and left palatine
12	Pterygoid apophysis
13	Anterior end of the edge of the foramen magnum
14	Posterior end of the edge of the foramen magnum
15	Point of crossing between the occipital condyle and the internal edge of the foramen magnum
16	Outermost point of the occipital condyle
17	Anterior tip of the external auditory meatus
18	Posterior tip of the zygomatic arch
19	Point of the rostrum furthest from the sagittal plane along the suture between the premaxilla and maxilla
Curve 1	Curve delimiting the snout, between landmarks 1 and 19 (8 semilandmarks)
Curve 2	Outer curve of the zygomatic arch, between landmarks 19 and 18 (18 semilandmarks)
Curve 3	Inner curve of the zygomatic arch, between landmarks 7 and 9 (18 semilandmarks)
Curve 4	Curve along the contour of the fourth upper premolar in a counterclockwise direction (18 semilandmarks)

To remove differences in position, scale, and orientation among the cranial configurations, we performed a Generalised Procrustes Analysis [15] sliding semilandmarks under the minimising bending energy criterion [48]. This analysis was carried out using the R package *geomorph* [3].

Centroid size was taken as the measure of the size of each cranium [14]. We log-transformed this variable so that it had a normal distribution. Finally, we calculate the mean shape and size of each species, which we used to carry out the statistical analysis.

In order to generate a morphospace of the cranium of flying squirrels, we performed a Principal Components Analysis (PCA) using the function “gm.prcomp” of the *geomorph* R package [3].

### Statistical analysis of cranial morphology

To analyse the effects of diet and cranial size on cranial shape, we performed a phylogenetic least squares analysis (PGLS) adapted to Procrustes variables [1]. We first tested the effect of diet on cranial size and then examined the effects of diet and cranial size on cranial shape, including the interaction term only if the first test was significant. PGLS is a phylogenetic comparative method that considers the phylogenetic relationships between species when analysing multivariate linear relationships [119]. For this analysis, we used the most recent phylogenetic tree published for the group [83], pruned to include only the species with cranial shape data. Cranial shape was represented by landmarks and semilandmarks (Procrustes coordinates), as the use of the PCA scores is discouraged [1]. We used “procD.pgls” function from the R package *geomorph* [2, 3], which performs a PGLS adapted to shape variables (Procrustes variables). It uses the Procrustes distance (square root of the sum of the squares of the distances between landmarks) between the expected and the observed shape instead of the covariances between variables to establish the statistical parameters (Sum of squares, F-values, or R-squared) [1]. To address the assumption of PGLS that residuals follow a Brownian motion model (with a phylogenetic signal of 1), we adjusted our phylogeny whenever this condition was not met. Specifically, we calculated Pagel’s lambda [92] for the residuals from a non-phylogenetic general linear model using the “procD.lm” function in the geomorph package and the “transformPhylo.ML” function in the motmot package [96]. Based on the estimated lambda, we modified the phylogeny by rescaling branch lengths using the “rescale” function in the geiger package [53] and conducted the PGLS analysis with the rescaled tree.

To test differences in variances of cranial shape among dietary categories, we performed pairwise comparisons of the dispersion around mean shapes using the “pairwise” function of the R package RRPP [29]. Additionally, we performed a PGLS to explore the impact of size alone on cranial shape, and then projected the PGLS shape regression scores on log centroid size to study the allometric variation of cranial shape.

### Disparity of shape and size within biomes

We estimated cranial shape disparity of species in each biome, defined as the Procrustes variance (Procrustes distance between the species and the biome mean shape). To do that, we used the function ShapeDist from the package *Evomorph* which uses Procrustes method for measuring distances between a group of shapes and a reference.

To identify the expected relationship between species richness and both shape and size disparity, we randomly drew 1,000 samples of flying squirrels varying in species richness from two to the maximum richness observed in the biomes (n = 26). Subsequently, we performed a regression analysis to examine whether disparity is dependent on species richness and determine whether the observed disparity in each biome significantly deviated from the expected distribution while maintaining consistent species numbers (i.e., the observed value was outside of the 95% of the expected distribution). For tropical biomes, evergreen equatorial rainforest (biome I) and tropical deciduous woodland (biome II), we tested if the observed disparity was higher than the expected, while for temperate and boreal biomes (biomes V, VI and VIII) we tested if the observed disparity was lower than the expected. Additionally, we conducted a reverse test to rule out the opposite hypothesis. We did this for both shape and size disparity.

All analyses were performed using the R programming language [97] with the aid of the previously cited packages and the *ggplot2* package for the generation of all plots [130], all analyses can be found in the supplementary material.

## Results

### Variations in cranial morphology of the flying squirrels

**Fig. 2:**
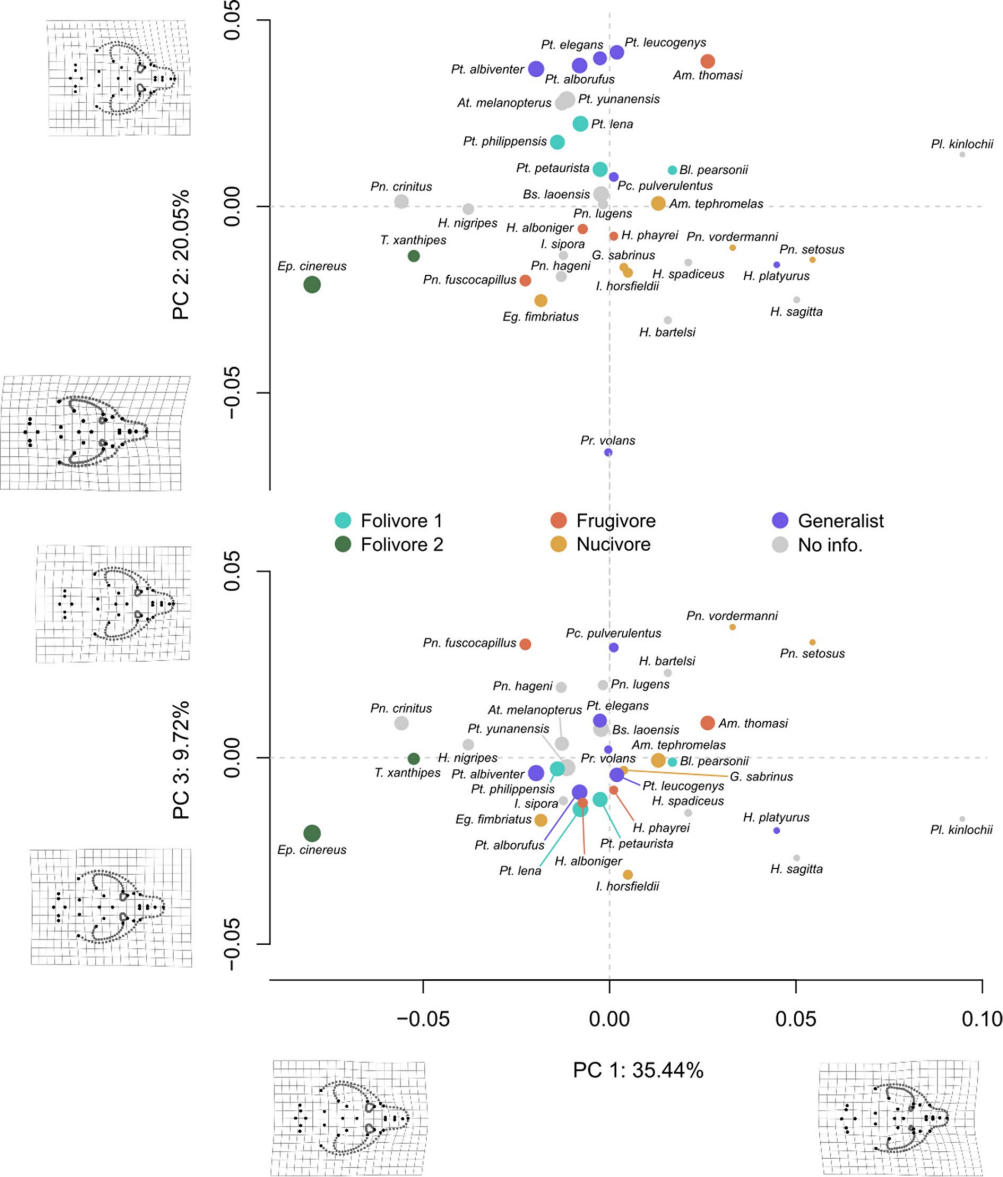
Cranial morphospace of the sampled species of the tribe Pteromyini (PC1 x PC2 shown above and PC1 x PC3 shown below). Dot size represents centroid size, and colour represents diet category (Table 2). Grids represent variations in cranial shape associated with the maximum and minimum values of each PC. Generas: *Aeretes* (*At.*), *Aeromys* (*Am.*), *Belomys* (*Bl.*), *Biswamoyopterus* (*Bs.*), *Eoglaucomys* (*Eg.*), *Eupetaurus* (*Ep.*), *Glaucomys* (*G.*), *Hylopetes* (*H.*), *Iomys* (*I.*), *Petaurillus* (*Pl.*), *Petaurista* (*Pt.*), *Petinomys* (*Pn.*), *Pteromys* (*Pr.*), *Pteromyscus* (*Pc.*), *Trogopterus* (*T.*)

The three first principal components (PCs) of the PCA defining the cranial morphospace of the flying squirrels explain 65.21% of the variation in cranial shape of the sampled squirrels (Fig. 2). PC1 accounts for 35.44% of this variation. Crania at the negative end of the axis are wide and elongated, with protruding and rounded zygomatic arches that frame a wide zygomatic fossa, an elongated snout, a wide P4, a long tooth row, and a very prominent masseter tubercle (Fig. 2). In contrast, crania at the positive end of the axis have a reduced P4, a shortened tooth row, a shortened snout and less protruding zygomatic arches. As a result, these crania have smaller zygomatic fossa (Fig. 2). Folivorous (type 2) species are situated towards the negative end of PC1 (Fig. 2) and nucivores towards the positive end. *Eupetaurus cinereus*, which feeds on acicular pine leaves [135], is located at the negative end of the PC1 axis, very close to *Trogopterus xanthipes*, a folivorous species that mainly feeds on oak and conifer leaves [129]. On the other hand, *Petinomys setosus*, which feeds on nuts and fruits [28, 89], is the species with a known diet closest to the positive end of the same axis.

PC2 accounts for 20.05% of the variation of the cranial shape (Fig. 2). At the positive end of the axis, species have short and rounded crania, with a very broad snout, large teeth, prominent masseter tubercle, rounded and protruding zygomatic arches, and a wide zygomatic fossa. In contrast, species at the negative end have elongated crania, with sharp snouts, small teeth, less protruding zygomatic arches and with a narrow and elongated zygomatic fossa. The positive section of the PC2 is occupied mainly by generalist and folivorous (type 1) species while other diets are located mainly towards negative values (Fig. 2). One notable exception is *Pteromys volans*, a generalist species that feeds mainly on leaves, nuts, and seeds [5], which displays a large divergence from the other negative-end species. PC3, in contrast, accounts for a small proportion of the sample’s morphological variation (9.72%). This axis separates crania with subtle differences in dentition size and snout roundness, with larger teeth and more rectangular shapes at the negative end (Fig. 2). In this axis, folivorous (type 1) and generalist squirrels are clustered around the centre of this axis, while nucivorous species diverge towards both ends.

### Influence of diet and size on cranial shape

**Fig. 3:**
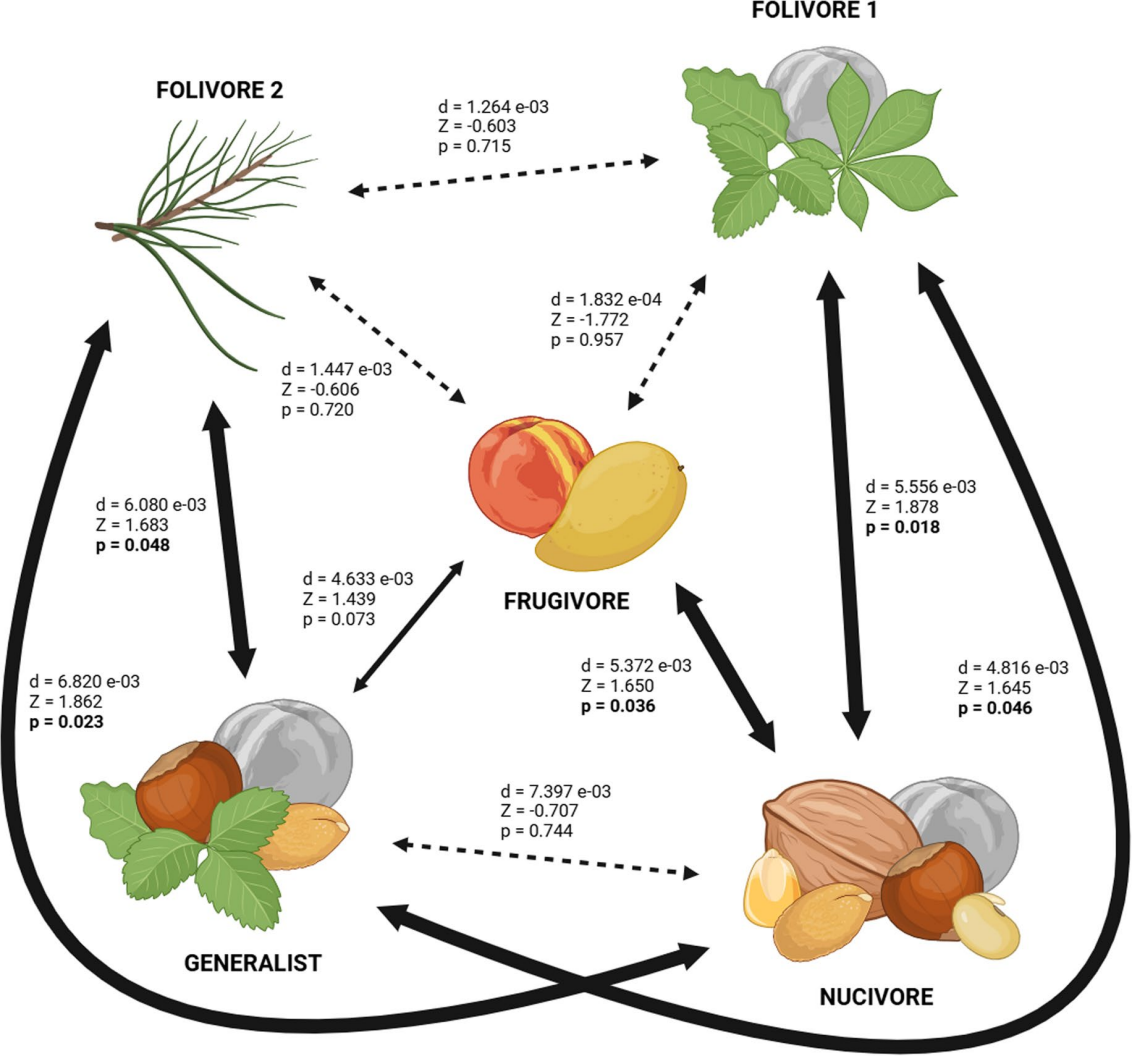
Pairwise comparisons of the variances of PGLS values for each diet. d, distance between variances; Z, effect size; p, significance value (significant differences are indicated in bold). Wide solid arrows connect diets that show significant differences, narrow solid arrows connect diets that show marginally significant differences, and narrow dashed arrows connect diets that do not show significant differences between them. Fruits shaded in gray indicate the possibility of being included in the diet despite not being considered in the categorization, as indicated in Table 2

The impact of diet and cranial size on cranial shape is statistically significant, explaining 29.5% and 12.8% of the variation in cranial shape respectively (Table 4). Pairwise comparisons between diets show that, despite their placement in different areas of the cranial morphospace (Fig. 2), both folivore diets do not show statistical differences in cranial shape between them, but they are significantly different from all the other diets, except frugivores, which, in turn, are only distinguishable from nucivores. Finally, there are no significant differences between nucivores and generalists either (Fig. 3).

**Table 4:** Results of Procrustes ANOVA/Procrustes PGLS analyses on the cranial morphology of squirrels of the tribe Pteromyini. Upper table: effect of diet on cranial size (centroid size). Middle table: effect of diet and cranial size (centroid size) on cranial shape. Lower table: effect of cranial size (centroid size) on cranial shape. Df, degrees of freedom; SS, sum of squares, MS, mean sum of squares; r^2^, coefficient of determination; p, significance value (significant p-values are indicated in bold)

Size ~ Diet	Df	SS	MS	r^2^	F	Z	p
Diet	4	0.009	2.2363 e-03	0.180	0.825	−0.066	0.528
Residuals	15	0.041	2.7106 e-03	0.820			
Total	19	0.050					
**Shape ~ Diet x Size**							
Diet	4	0.001	1.5399 e-04	0.295	2.032	2.383	**0.010**
Size	1	0.000	2.6678 e-04	0.128	3.521	2.817	**0.002**
Residuals	14	0.001	7.58 e-05	0.507			
Total	19	0.002					
**Shape ~ Size**							
Size	1	0.000	4.0314 e-04	0.193	4.298	3.055	**0.001**
Residuals	18	0.002	9.379 e-05	0.807			
Total	19	0.002

**Fig. 4:**
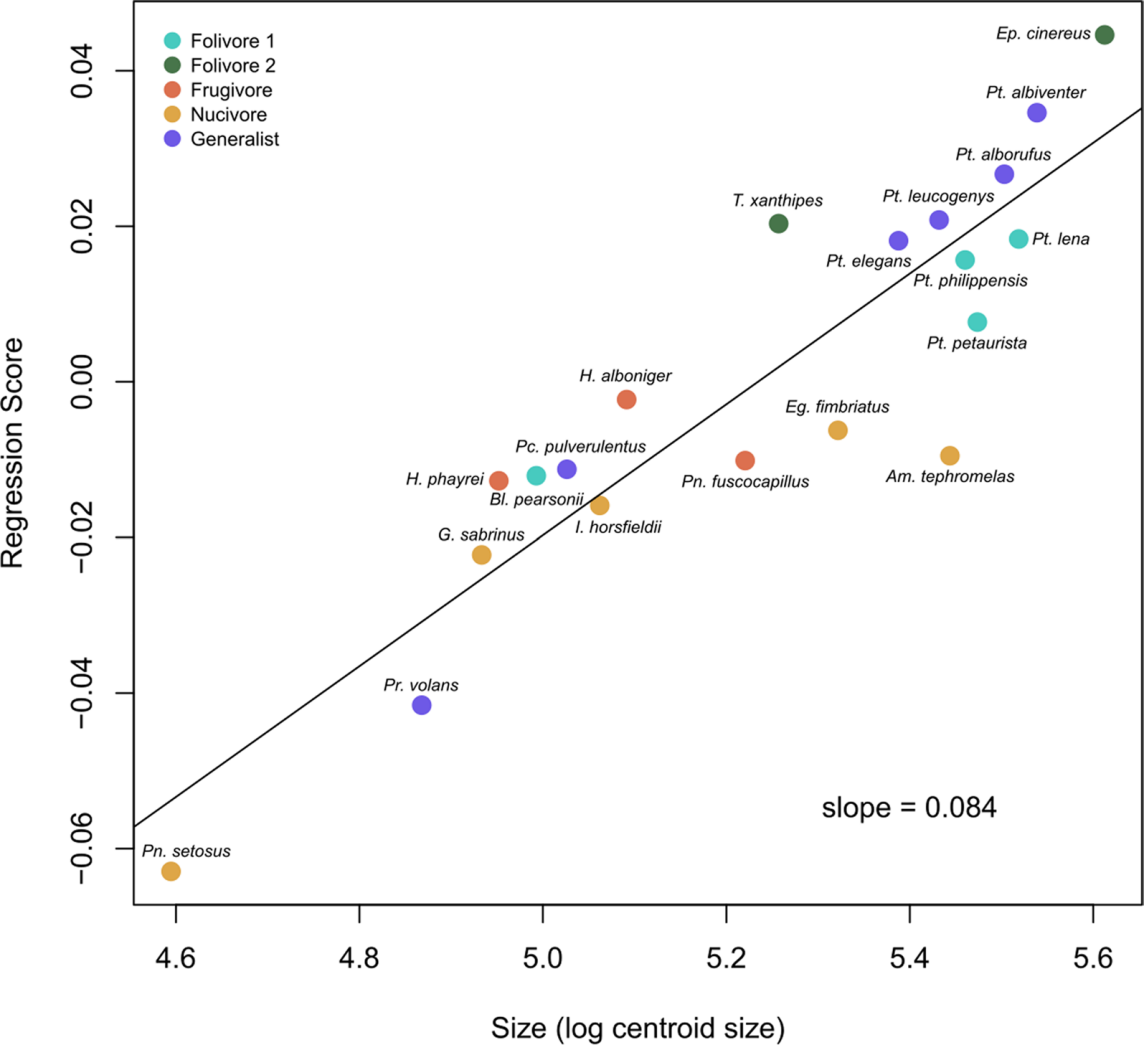
Allometric relationship between cranial shape (PGLS regression scores) with cranial size of the sampled species of the tribe Pteromyini. Colours represent dietary categories (Fig. 2). Generas: *Aeromys* (*Am.*), *Belomys* (*Bl.*), *Eoglaucomys* (*Eg.*), *Eupetaurus* (*Ep.*), *Glaucomys* (*G.*), *Hylopetes* (*H.*), *Iomys* (*I.*), *Petaurista* (*Pt.*), *Petinomys* (*Pn.*), *Pteromys* (*Pr.*), *Pteromyscus* (*Pc.*), *Trogopterus* (*T.*)

Regarding the impact of cranial size on cranial shape, PGLS regression of cranial shape on log-transformed centroid size is significant (Table 4), indicating the existence of allometric variation (Fig. 4). Folivorous and generalist species, which have the widest snouts, large teeth in a long tooth row, and a very prominent masseter tubercle (Fig. 2), have the largest crania (Fig. 4). The species with medium cranium size feed mainly on fruits and nuts (Fig. 4), and they have shorter and wider crania, with protruding zygomatic arches, a relatively wide zygomatic fossa, and small teeth in a short tooth row (Fig. 2). However, nucivorous species have a wide range of sizes (Fig. 4). The species with the smallest crania are *Petinomys setosus* and *Pteromys volans* (Fig. 4), a nucivore and generalist species, respectively, with extreme cranial shapes (Fig. 2).

### Cranial shape and size disparity across biomes

**Fig. 5:**
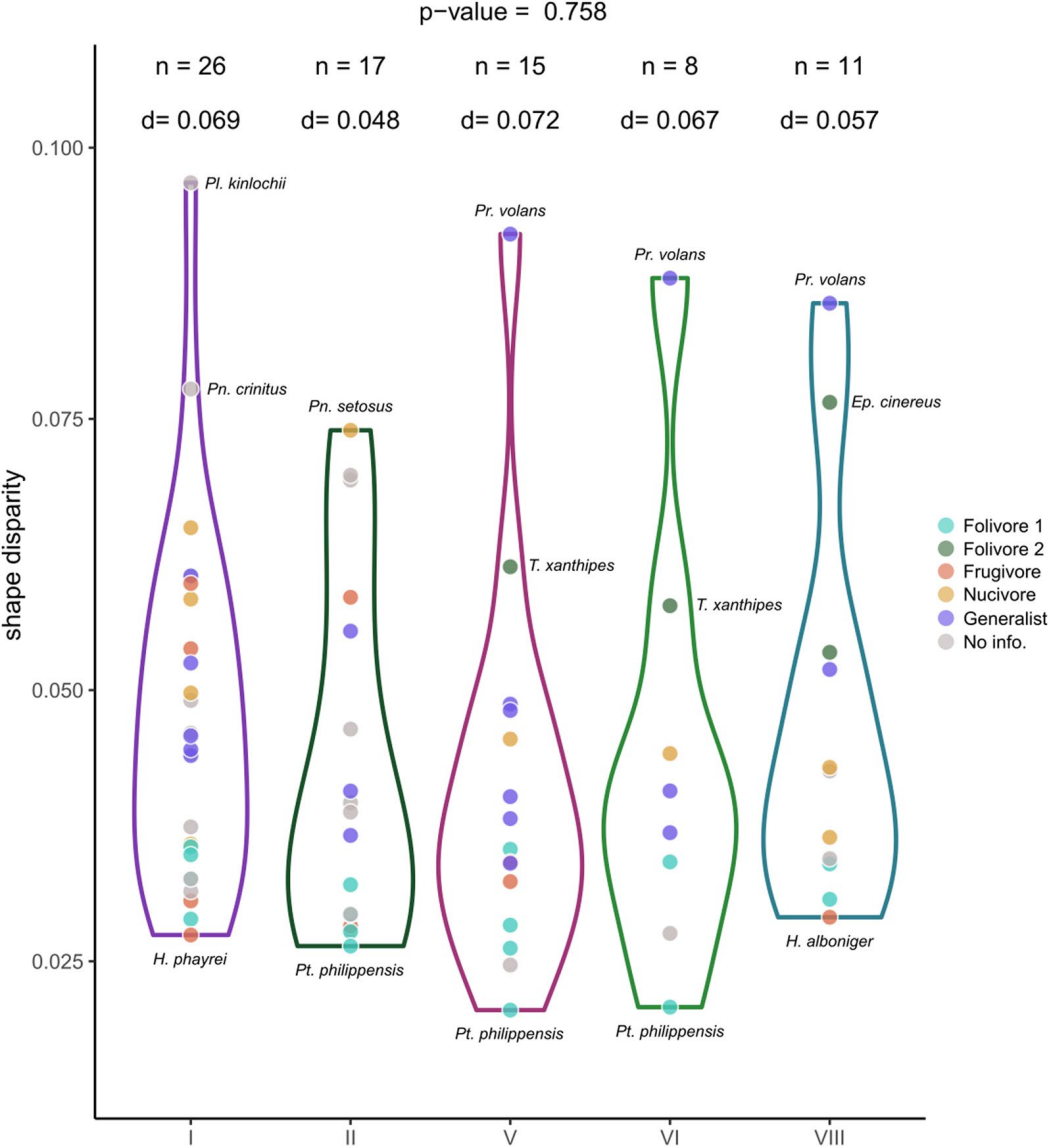
Shape disparity of species by biomes. Dots represent species present in each biome coloured by their diet. Y axis represent Procrustes distance to the biome mean. N is the number of species present in each biome. D is the total disparity for each biome. Biomes: evergreen equatorial rainforest (I), tropical deciduous woodland (II), temperate evergreen forest (V), broad-leaf deciduous forest (VI), taiga (VIII). Genus: *Eupetaurus* (*Ep.*), *Hylopetes* (*H.*), *Petaurillus* (*Pl.*), *Petaurista* (*Pt.*), *Petinomys* (*Pn.*), *Pteromys* (*Pr.*), *Trogopterus* (*T.*)

**Fig. 6:**
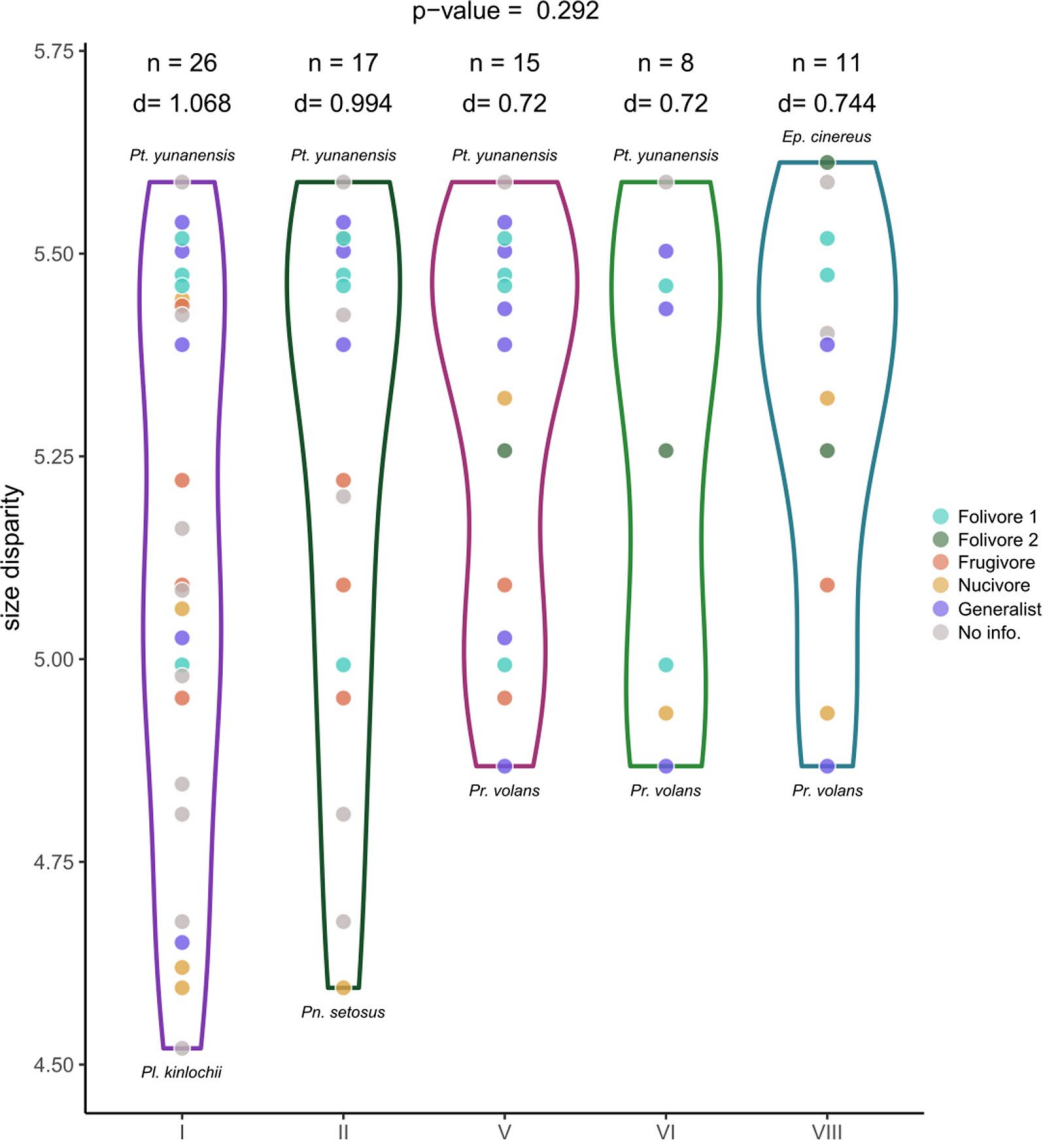
Size disparity of species by biomes. Dots represent species present in each biome colored by their diet. Y axis represent Procrustes distance to the mean centroid size. N is the number of species present in each biome. D is the total disparity for each biome. Biomes: evergreen equatorial rainforest (I), tropical deciduous woodland (II), temperate evergreen forest (V), broad-leaf deciduous forest (VI), taiga (VIII). Genus: *Eupetaurus* (*Ep.*), *Petaurillus* (*Pl.*), *Petaurista* (*Pt.*), *Petinomys* (*Pn.*), *Pteromys* (*Pr.*)

Regarding shape disparity (Fig. 5), the biome with the greatest disparity is the temperate evergreen forest (V), followed by evergreen equatorial rainforest (I), broad-leaf deciduous forest (VI), taiga (VIII), and tropical deciduous woodland (II). However, we found no statistical differences in shape disparity among biomes (ANOVA, p = 0.758). On the other hand, regarding size disparity, the evergreen equatorial rainforest (I) is the biome with the greatest size disparity, followed by tropical deciduous woodland (II), taiga (VIII), and broad-leaf deciduous forest (VI) and temperate evergreen forest (V), with the same size disparity (Fig. 6). We also found no significant differences among biomes for size disparity (ANOVA, p = 0.292).

**Fig. 7:**
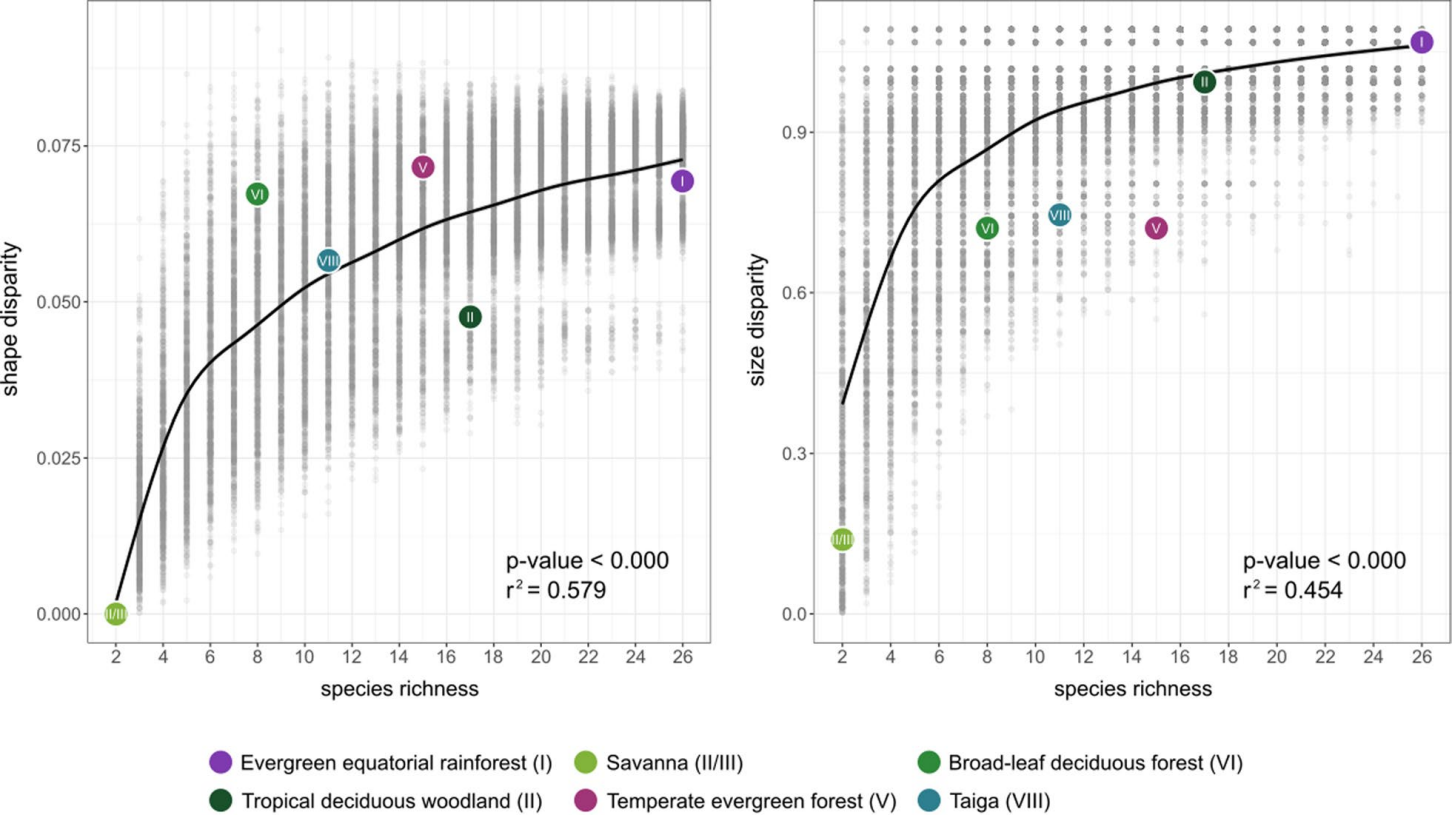
Regression of shape (left) and size (right) with species richness in 1000 permutations. Coloured dots indicate the observed values for each biome

**Fig. 8:**
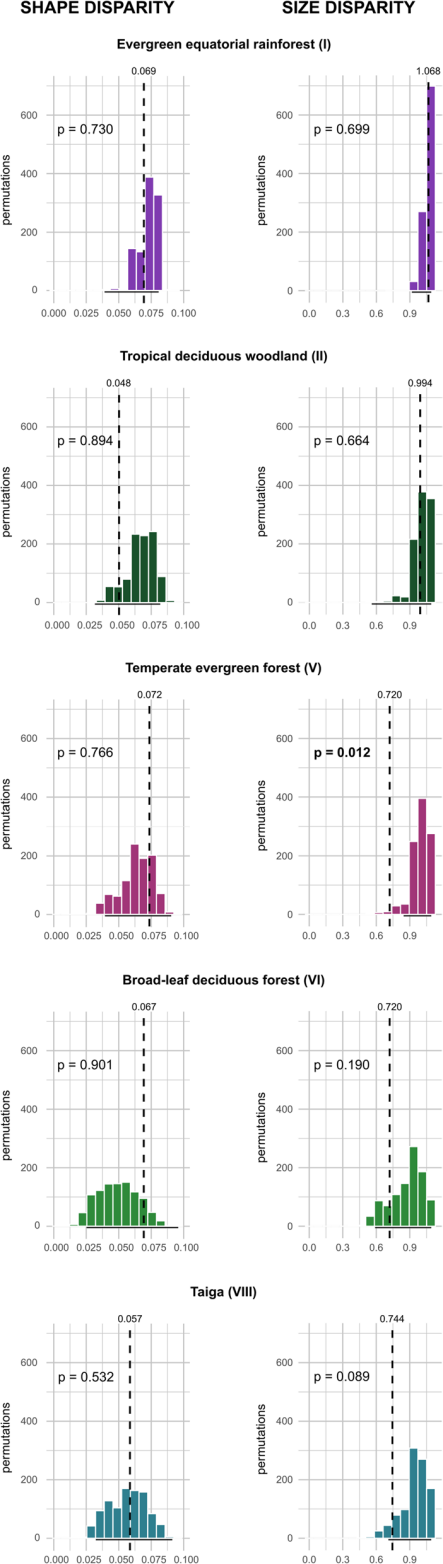
Observed and simulated shape (left) and size (right) disparity for the different biomes. Bars represent the simulated frequencies of shape and size disparity based on species richness in each biome. The horizontal black line shows the 95% confidence interval. The vertical dashed line represents the observed value. p, probability in each biome that the observed morphological disparity is different (higher or lower) than the estimated based on species richness

Our simulations show the expected shape and size disparity increase with species richness (Fig. 7). When plotting the observed values of shape disparity of each biome in the simulated relationship we observe that evergreen equatorial rainforest (biome I), savannah (biome II/III), and taiga (biome VIII) have a disparity similar to the expected regression, while that tropical deciduous woodland (biome II) shows lower, and the temperate biomes (V, VI) show higher disparity. As for size disparity, tropical biomes show similar values to the expected regression, but temperate biomes and savannah have lower disparity. However, when tested, we did not find statistical differences of the observed shape and size disparity with the null distribution based on the simulations, except for biome V that showed a significantly lower size disparity than estimated for its species richness and biome VIII shows a marginal statistical significant effect (p = 0.089) in size disparity (Fig. 8). That is, in general the observed disparity is not different than expected for the species richness in each biome.

## Discussion

### Relationship between cranial shape, diet, and cranial size

Diet has an overall impact in cranial shape in flying squirrels, explaining 36% of the variation in cranial morphology (Table 4), as we can see in other rodents [22, 93, 104]. Additionally, although to a lesser extent, cranial shape also undergoes significant modifications as a function of cranial size (Table 4. These variations in cranial shape and size appear to be mostly associated to differences in the insertion and space requirements of the muscles involved in mastication (deep and superficial masseters as well as zygomaticomandibularis among species with different diets [32, 123].

For example, nucivorous species, which feed mainly on nuts and seeds, need for powerful jaw muscles to open nuts by gnawing with the incisors may be favouring the relative width of the cranium in certain cases, as for example in *Petinomys setosus* and *Petinomys vordermanni* (Fig. 2). On the other hand, frugivorous species do not seem to have any tendency towards certain cranial shapes, but have medium cranial shapes, since a fruit-based diet does not require such powerful jaw muscles. However, as has been seen in frugivorous marsupials, bats and primates, a differential preference of species for specific fruits with particular physical properties could determine cranial shape [13, 39]. For example, *Hylopetes alboniger*, which has medium cranial shape, feeds on different ripened fruits depending on the season, such as *Psidium guajava*, *Neolamarckia cadamba* and *Ficus curtipes* fruits, among others [68]. On the other hand, *Petinomys fuscocapillus* feeds on fruits from more than 10 different plant species but has a preference for *Hydnocarpus pentandra* fruits [23], which could condition its cranial shape somewhat further from the mean values of the morphospace. Finally, *Aeromys thomasi* has a larger, wider and more robust shape, more similar to the folivorous and generalist species (Fig. 2). This could be due to the limited ecological knowledge that exists about this species, since, although it has been described as mainly frugivorous [46] the most recent observations suggest that it could have a more generalist diet [34].

The robust and protruding zygomatic arches of folivorous species (type 1 and 2) allows for the insertion and passage of large and strong muscles, required for a diet based on hard and abrasive plant matter [104, 106]. They also have a prominent masseter tubercle, which provides a larger insertion point for the superficial masseter, one of the muscles that is most involved in mastication in squirrels [32]. The large insertion surface of the chewing muscles in folivorous species is consistent with previous studies in squirrel jaws, which showed that folivorous and herbivorous squirrel species have a mandible with deeper and longer angular process than other squirrels, as well as a reduced coronoid process [21, 118, 137]. This shape is associated with an increase in the relative bite force [118], since it increases the insertion area of the superficial and deep masseters. In addition, not only the diet determines the mandible shape but also the size, independently and jointly [137], as in the case of cranial shape.

Additionally, differences in cranial morphology among dietary groups are also associated to the dentition. For example, folivores exhibit a wide snout that houses a set of large teeth adapted to support this abrasive diet. Folivory generates high dental wear due to both the abrasive nature of cellulose and the low nutritional value of leaves, which require prolonged times of mastication [73, 137]. The necessary energy for a folivorous diet may be compensated through the increase in body size, decreasing their metabolic rate in relation to their body size [82, 107]. Modifications of cranial shape and size such as the ones observed here for the folivorous species of flying squirrels are even more pronounced in other folivorous groups of squirrels (e.g. marmots), whose diet includes grasses that are more abrasive than other types of leaves [131]. Type 2 folivores have a very abrasive diet. On the one hand, *Eupetaurus cinereus* has an extreme cranial morphology (Fig. 2), and the largest size of all the species analysed (Fig. 4). Additionally, it has developed hypsodont teeth [135] that are larger than expected for its body size [84]. These adaptations are likely related to the hardness and low nutritional value of a diet specialised on conifer needles [51], which requires consuming large amounts of them and causes rapid tooth wear. On the other hand, *Trogopterus xanthipes* also feeds on abundant conifer needles, although its diet also includes oak leaves [129]. The influence of hypsodonty in both species could also explain the differences in shape between them and type 1 folivores (Fig. 2). The development of hypsodonty in other groups of mammals is a well-known process, especially related to the consumption of grasses, which are also abrasive foods with low nutritional intake [76].

Finally, generalist species mostly have medium shapes in PC1 and PC3 but an extreme cranial shape in PC2 (Fig. 2), as well as large cranial sizes (Fig. 4), with the exception of *Pteromyscus pulverulentus*, which has medium shape and size. These extreme morphologies differ with the moderate morphological characters found in other omnivorous rodents (i.e. *Rattus rattus*), with intermediate shapes between herbivores and insectivores and carnivores [104]. *Petaurista albiventer*, *P. alborufus*, *P. elegans* and *P. leucogenys* have large, wide and rounded crania (Figs. 2 and 4), which could be explained by the predominance of leaves and nuts in their diet, since they need large and strong muscles not only for processing abrasive plant matter but also for opening nuts. A similar pattern has been observed in platyrrhine primates, with omnivorous species having relatively robust skulls and large teeth [8], in prairie dogs, which have extreme dental morphologies related to a diet that includes grass and dry fruits [84], and in ungulates, with omnivorous species having higher bite forces [94]. On the other hand, *Hylopetes platyurus*, that feeds on leaves, fruit, nuts and insects [47, 89], has one of the smallest and shortest cranium (Fig. 2). Regarding *Pteromys volans*, it has a small cranium (Fig. 4) and the narrowest and elongated one (Fig. 2), being the only species with these characteristics, associated with an insectivorous diet in other rodents [56, 77, 104]. Although some foods eaten by this flying squirrel are hard and abrasive (i.e. pine needles, nuts), *P. volans* tends to avoid the harder parts of foods, for example, eating only the mesophyll of the pine needles, buds instead of mature leaves, pine-seeds only at early stages of ripening, or avoiding leave veins [5], which could explain its narrow cranium.

Despite the observable differences in shape between diets (Fig. 2), pairwise comparisons between them show that nucivores and generalists are the only diet with a significantly different cranial shape from the other diets, but not from each other (Fig. 3). All generalist species included in this study feed on nuts, so this suggests that their presence in the diet would have a great impact on the cranial shape of flying squirrels.

### Shape and size disparity by biomes

Under the hypothesis of greater disparity in biomes with more niches available we would expect higher disparity in tropical biomes. However, we found no statistical differences in shape disparity among tropical and temperate biomes. In fact, despite the fact that tropical biomes (biomes I and II) have a greater number of squirrel species than temperate biomes (Table 1), we found that the temperate evergreen forest (biome V) has slightly higher shape disparity. Also, although not statistically significant, temperate biomes showed relative high shape disparity above the regression line of the expected disparity for their species richness (Fig. 7), while evergreen equatorial rainforest showed a more similar value to the regression line. Therefore, it appears that tropical biomes have greater disparity in shape just because they have more species, as we did not find more disparity than expected based on their species richness. These results are not consistent with the hypothesis of greater disparity of shape adaptations due to niche packing in tropical biomes. The relatively high disparity in the temperate biomes is mostly produced by the presence of species that feed on pine needles and other harsh leaves, such as *Eupetaurus cinereus*, *Trogopterus xanthipes* and *Pteromys volans* [5, 129, 134, 135]. These species have extreme cranial shapes, related to the functional demands of their diets, which are extremely abrasive and low nutritional [51]. This suggests that the number of available niches might not be determinant in producing high morphological disparity, but the existence of extreme niches which require specific adaptations to exploit them (i.e., folivory) might be more important.

As for the cranial size, we found the opposite pattern, where temperate biomes showed relative lower size disparity compared to the regression line (Fig. 7). This greater disparity in size but not in shape in tropical biomes could be due to the fact that niche partitioning in tropical biomes would occur in size and not in shape, since it is more evolutionarily costly. However, the results also show no significant differences according to the simulations, except for the temperate evergreen forest (biome V), which shows values below the simulated distribution for their species richness (Fig. 8).

The low disparity in size in temperate biomes is mostly due to the absence of an important guild of small species like some species from *Hylopetes*, the dwarf flying squirrels (*Petinomys*), and the pygmy flying squirrels (*Petaurillus*). This could be explained by the relationship that have been observed between the body mass of flying squirrels and glide ratio and distance [36, 62], according to which, the larger their size, the greater the horizontal distance they reach and the greater the glide ratio they have [36, 134]. Furthermore, larger species may achieve higher speeds during gliding, which, coupled with longer glides, would result in less locomotor control during gliding [11, 133]. Because of this, larger species would be better adapted to more open forests, being able to glide longer distances, while smaller species would be better adapted to the middle forest strata (subcanopy and understory), performing shorter and more manoeuvrable glides [116, 117, 122]. This is consistent with a greater presence of small flying squirrels in tropical biomes, as these forests are dominated by trees, lianas and large shrubs, making the mid-upper forest strata (understory, subcanopy and canopy) more dense than in temperate forests [113]. In addition, tree growth in tropical biomes is more clumped, while a more random spatial pattern predominates in temperate forests [9], increasing the required glide horizontal distances.

## Conclusions

Cranial shape of flying squirrels (tribe Pteromyini) is highly related with diet through the interaction between food properties and the structure of the masticatory muscular apparatus necessary to process it. A more abrasive and less nutritive diet, as is the case of the folivorous species, requires more powerful masticatory muscles. As a result, the cranium of these species is wider, with wide zygomatic arches, a wide zygomatic fossa, and a prominent masseter tubercle that allows the accommodation of larger muscles. Additionally, there is an allometric pattern, with cranial size independently influencing its shape. This relationship is likely due to the structural requirements (larger teeth) and energy demands (higher consumption and longer shewing time) involved in processing this type of food. Species with more nutritious diets, such as nucivorous or frugivorous, are associated with smaller crania and/or more gracile forms.

Considering that there is a relationship between cranial morphology and diet, we would expect that there would be more morphological disparity in those biomes with more different dietary niches. However, we did not find differences in cranial morphological disparity among tropical and temperate biomes. Tropical biomes appear to have more disparity due to a higher number of species, but this disparity is not greater than what would be expected based on species richness alone. This might indicate that the existence of extreme ecological niches that require specialised adaptations for their exploitation may be more important for generating high morphological disparity than only the number of available niches alone. Moreover, adaptation to different niches by changing body size instead of shape adaptations could also mitigate the amount of shape disparity in biomes with high number of available resources.

## Electronic supplementary material

Below is the link to the electronic supplementary material.

Supplementary file 1 (XLSX 140 kb)

## Data Availability

The datasets used and analysed during the current study are available from the corresponding author on reasonable request.

## References

[1] Adams DC. A method for assessing phylogenetic least squares models for shape and other high-dimensional multivariate data. Evolution. 2014;68:2675–88. 10.1111/evo.12463. 24899536 10.1111/evo.12463

[2] Adams DC, Collyer M. Permutations test for phylogenetic comparative analyses of high-dimensional shape data: what you shuffle matters. Evolution. 2015;69:823–9. 10.1111/evo.12596. 25641367 10.1111/evo.12596

[3] Adams DC, Collyer M, Kaliontzopoulou A, Baken, E. Geomorph: Software for geometric morphometric analyses. R package version 4.0.4. 2022.

[4] Adams D, James Rohlf F, Slice DE. Geometric morphometrics: ten years of progress following the ‘revolution.’ Ital J Zoo. 2004;71:5–16. 10.1080/11250000409356545.

[5] Airapetyants AE, Fokin IM. Biology of European flying squirrel *Pteromys* volans L. (Rodentia: Pteromyidae) in the North-West of Russia. Russ J Theriol. 2003;2:105–13.

[6] Allué Andrade JL. Atlas fitoclimático de España: taxonomías. Madrid: INIA; 1990.

[7] Amori G, Gippoliti S, Luiselli L, Battisti C. Are there latitudinal gradients in taxa turnover? A worldwide study with Sciuridae (Mammalia: Rodentia). Community Ecol. 2010;11:22–6. 10.1556/comec.11.2010.1.4.

[8] Anapol F, Lee S. Morphological adaptation to diet in Platyrrhine Primates. Am J Phys Anthropol. 1994;94:239–61. 10.1002/ajpa.1330940208. 8085615 10.1002/ajpa.1330940208

[9] Armesto JJ, Mitchell JD, Villagran C. A comparison of spatial patterns of trees in some tropical and temperate forests. Biotropica. 1986;18:1–11. 10.2307/2388354.

[10] Baverstock H, Jeffery NS, Cobb SN. The morphology of the mouse masticatory musculature. J Anat. 2013;223:46–60. 10.1111/joa.12059. 23692055 10.1111/joa.12059 PMC4487762

[11] Berghäuser T, Nyakatura JA, Wölfer J. Evolution of gliding in squirrel-related rodents (Mammalia: Sciuromorpha) did not induce a new optimum on the cortical thickness of the scapular glenoid fossa. Anat Rec. 2022;306:2716–28. 10.1002/ar.25146. 10.1002/ar.25146 36583480

[12] Black CC. Holarctic evolution and dispersal of squirrels (Rodentia: Sciuridae). In: Dobzhansky T, Hecht MK, Steere WC, editors. Evolutionary biology. New York: Springer; 1972. p. 305-322. 10.1007/978-1-4684-9063-3_10

[13] Bonaccorso FJ, Gush TJ. Feeding behaviour and foraging strategies of captive phyllostomid fruit bats: an experimental study. J Anim Ecol. 1987;56:907–20. 10.2307/4956.

[14] Bookstein FL. “Size and shape”: a comment on semantics. Syst Zool. 1989;38:173–80. 10.2307/2992387.

[15] Bookstein FL. Morphometric tools for landmark data: Geometry and biology. Cambridge: Cambridge University Press; 1991.

[16] Bookstein FL. Combining the tools of geometric morphometrics. In: Marcus LF, Corti M, Loy A, Naylor GJP, Slice DE, editors. Advances in morphometrics. Boston: Springer; 1996. p. 131-151. 10.1007/978-1-4757-9083-2_12

[17] Buckley LB, Jonathan Davies T, Ackerly DD, Kraft NJB, Harrison SP, Anacker BL, Cornell HV, Damschen EI, Grytnes J-A, Hawkins BA, McCain CM, Stephens PR, Wiens JJ. Phylogeny, niche conservatism and the latitudinal diversity gradient in mammals. Proc R Soc B. 2010;277:2131–8. 10.1098/rspb.2010.0179. 20335205 10.1098/rspb.2010.0179 PMC2880153

[18] Bukombe J, Kittle A, Senzota RB, Kija H, Mduma S, Fryxell JM, Magige F, Mligo C, Sinclair ARE. The influence of food availability, quality and body size on patch selection of coexisting grazer ungulates in western Serengeti national park. Wildl Res. 2019;46:54–63. 10.1071/WR18072.

[19] Cardini A, O’Higgins P. Patterns of morphological evolution in *Marmota* (Rodentia, Sciuridae): geometric morphometrics of the cranium in the context of marmot phylogeny, ecology and conservation. Biol J Linn Soc. 2004;82:385–407. 10.1111/j.1095-8312.2004.00367.x.

[20] Cardini A, O’Higgins P. Post-natal ontogeny of the mandible and ventral cranium in *Marmota* species (Rodentia, Sciuridae): allometry and phylogeny. Zoomorphology. 2005;124:189–203. 10.1007/s00435-005-0008-3.

[21] Casanovas-Vilar I, van Dam J. Conservatism and adaptability during squirrel radiation: what is mandible shape telling us? PLoS ONE. 2013;8:e61298. 10.1371/journal.pone.0061298. 23593456 10.1371/journal.pone.0061298 PMC3617180

[22] Caumul R, Polly PD. Phylogenetic and environmental components of morphological variation: skull, mandible, and molar shape in marmots (*Marmota*, Rodentia). Evolution. 2005;59:2460–72. 10.1111/j.0014-3820.2005.tb00955.x. 16396186

[23] Chakraborty R. An account small travancore flying squirrel, *Petinomys fuscocapillus fuscocapillus* (Jerdon). Rec Zool Surv India. 2008;108:33–48.

[24] Chartier M, von Balthazar M, Sontag S, Löfstrand S, Palme T, Jabbour F, Sauquet H, Schönenberger J. Global patterns and a latitudinal gradient of flower disparity: perspectives from the angiosperm order Ericales. New Phytol. 2021;230:821–31. 10.1111/nph.17195. 33454991 10.1111/nph.17195 PMC8048689

[25] Chown SL, Gaston KJ. Areas, cradles and museums: the latitudinal gradient in species richness. Trends in Ecol Evol. 2000;15:311–5. 10.1016/S0169-5347(00)01910-8. 10.1016/s0169-5347(00)01910-8 10884694

[26] Clauss M, Schwarm A, Ortmann S, Streich WJ, Hummel J. A case of non-scaling in mammalian physiology? Body size, digestive capacity, food intake, and ingesta passage in mammalian herbivores. Comp Biochem Physiol. 2007;148:249–65. 10.1016/j.cbpa.2007.05.024. 10.1016/j.cbpa.2007.05.024 17643330

[27] Clauss M, Steuer P, Müller DWH, Codron D, Hummel J. Herbivory and body size: allometries of diet quality and gastrointestinal physiology, and implications for herbivore ecology and dinosaur gigantism. PLoS ONE. 2013;8:e68714. 10.1371/journal.pone.0068714. 24204552 10.1371/journal.pone.0068714 PMC3812987

[28] Clayton E. *Petinomys setosus*. The IUCN Red List of Threatened Species 2016. 2016;e.T16739A22241609.

[29] Collyer ML, Adams DC. RRPP: an R package for fitting linear models to high-dimensional data using residual randomization. Methods Ecol Evol. 2018;9:1772–9. 10.1111/2041-210X.13029.

[30] Colwell RK, Hurtt GC. Nonbiological gradients in species richness and a spurious rapoport effect. Am Nat. 1994;144:570–95. 10.1086/285695.

[31] Cox PG, Jeffery N. The muscles of mastication in rodents and the function of the medial pterygoid. In: Cox P, Hautier L, editors. Evolution of the Rodents: Advances in Phylogeny, Functional Morphology and Development. Cambridge: Cambridge University Press; 2015. p. 350–72.

[32] Cox PG, Watson PJ. Masticatory biomechanics of red and grey squirrels (*Sciurus vulgaris* and *Sciurus carolinensis*) modelled with multibody dynamics analysis. R Soc Open Sci. 2023;10:220587. 10.1098/rsos.220587. 36816846 10.1098/rsos.220587 PMC9929510

[33] Dean MN, Bizzarro JJ, Summers AP. The evolution of cranial design, diet, and feeding mechanisms in batoid fishes. Integr Comp Biol. 2007;47:70–81. 10.1093/icb/icm034. 21672821 10.1093/icb/icm034

[34] Dehling JM. New dietary records for the rare Thomas’s flying squirrel (*Aeromys thomasi*, Sciuridae: Pteromyini) from Sabah. Malays Borneo Mammalia. 2024;88:98–9. 10.1515/mammalia-2023-0159.

[35] Demment MW, Van Soest PJ. A nutritional explanation for body-size patterns of ruminant and nonruminant herbivores. Am Nat. 1985;125:641–72. 10.1086/284369.

[36] Dial R. Energetic savings and the body size distributions of gliding mammals. Evol Ecol Res. 2003;5:1151–62.

[37] Donahue CA, Traxler KM, Walters KR, Louer JW, Gilford MT, Edwards ME, Harding JL, Bonam RC, Straw SA. Equatorial Africa: A Climatological Study. Scott AFB; 1995.

[38] Dubay SA, Hayward GD, Martínez del Rio C. Nutritional value and diet preference of arboreal lichens and hypogeous fungi for small mammals in the rocky mountains. Can J Zool. 2008;86:851–62. 10.1139/Z08-054.

[39] Dumont ER. Cranial shape in fruit, nectar, and exudate feeders: implications for interpreting the fossil record. Am J of Phys Anthropol. 1997;102:187–202. 9066900 10.1002/(SICI)1096-8644(199702)102:2<187::AID-AJPA4>3.0.CO;2-W

[40] Economo EP, Narula N, Friedman NR, Weiser MD, Guénard B. Macroecology and macroevolution of the latitudinal diversity gradient in ants. Nat Commun. 2018;9:1778. 10.1038/s41467-018-04218-4. 29725049 10.1038/s41467-018-04218-4 PMC5934361

[41] Felice RN, Tobias JA, Pigot AL, Goswami A. Dietary niche and the evolution of cranial morphology in birds. Proc R Soc B. 2019;286:20182677. 10.1098/rspb.2018.2677. 30963827 10.1098/rspb.2018.2677 PMC6408879

[42] Field R, Hawkins BA, Cornell HV, Currie DJ, Diniz-Filho JAF, Guégan J-F, Kaufman DM, Kerr JT, Mittelbach GG, Oberdorff T, O’Brien EM, Turner JRG. Spatial species-richness gradients across scales: a meta-analysis. J Biogeogr. 2008;36:132–47. 10.1111/j.1365-2699.2008.01963.x.

[43] Fischer AG. Latitudinal variations in organic diversity. Evolution. 1960;14:64–81. 10.2307/2405923.

[44] Freeman PW. Frugivorous and animalivorous bats (Microchiroptera): dental and cranial adaptations. Biol J Linn Soc. 1988;33:249–72. 10.1111/j.1095-8312.1988.tb00811.x.

[45] Fuhrman JA, Steele JA, Hewson I, Schwalbach MS, Brown MV, Green JL, Brown JH. A latitudinal diversity gradient in planktonic marine bacteria. PNAS. 2008;105:7774–8. 10.1073/pnas.0803070105. 18509059 10.1073/pnas.0803070105 PMC2409396

[46] Gerrie R, Kennerley R, Koprowski J. *Aeromys thomasi* (errata version published in 2017). The IUCN Red List of Threatened Species 2016. 2016;e.T557A115050074.

[47] Gerrie R, Kennerley R, Koprowski J. *Hylopetes platyurus*. The IUCN Red List of Threatened Species 2019. 2019;e.T136262A22244459.

[48] Green WDK. The thin-plate spline and images with curving features. In: Mardia KV, Gill CA, Dryden IL, editors. Image fusion and shape variability. Leeds: University of Leeds Press; 1996. p. 79–87.

[49] Gunz P, Mitteroecker P. Semilandmarks: a method for quantifying curves and surfaces. Hystrix It J Mamm. 2013;24:103–9. 10.4404/hystrix-24.1-6292.

[50] Hampton PM. Comparison of cranial form and function in association with diet in natricine snakes. J Morphol. 2011;272:1435–43. 10.1002/jmor.10995. 21780158 10.1002/jmor.10995

[51] Han W, Fang J, Guo D, Zhang Y. Leaf nitrogen and phosphorus stoichiometry across 753 terrestrial plant species in China. New Phytol. 2005;168:377–85. 10.1111/j.1469-8137.2005.01530.x. 16219077 10.1111/j.1469-8137.2005.01530.x

[52] Hanya G, Stevenson P, van Noordwijk M, Wong ST, Kanamori T, Kuze N, Aiba S-I, Chapman CA, van Schaik C. Seasonality in fruit availability affects frugivorous primate biomass and species richness. Ecography. 2011;34:1009–17. 10.1111/j.1600-0587.2010.06775.x.

[53] Harmon LJ, Weir JT, Brock CD, Glor RE, Challenger W. GEIGER: investigating evolutionary radiations. Bioinformatics. 2008;24:129–31. 10.1093/bioinformatics/btm538. 18006550 10.1093/bioinformatics/btm538

[54] Harrison JL. The natural food of some Malayan mammals. Bull Singapore Nat Mus. 1962;30:5–18.

[55] Hawkins BA, Porter EE, Felizola Diniz-Filho JA. Productivity and history as predictors of the latitudinal diversity gradient of terrestrial birds. Ecology. 2003;84:1608–23.

[56] Hennekam JJ. Comparative morphology of the dormouse skull and the influence of size and ecology. J Anat. 2022;240:914–35. 10.1111/joa.13596. 34784427 10.1111/joa.13596 PMC9005685

[57] Hernández Fernández M. Bioclimatic discriminant capacity of terrestrial mammal faunas. Glob Ecol Biogeogr. 2001;10:189–204. 10.1046/j.1466-822x.2001.00218.x.

[58] Hernández Fernández M, Pelegrin JS, Gómez Cano AR, García Yelo BA, Moreno-Bofarull A, Sánchez-Fontela N, Rodríguez-Ruiz C, Camacho AR, Domingo L, Menéndez I, Martín-Perea DM, Bazán CM, Alcalde GM, Domingo MS, Luna B, Peinado Cortés MM, Arias A, González Couturier G, Márquez Villena A, Anaya N, Blanco F, Galli E, Gamboa S, Quesada Á, Sanz-Pérez D, Varela S, Cantalapiedra JL. Macroevolution and climate changes: a global multi-family test supports the resource-use hypothesis in terrestrial mammals. Hist Biol. 2022;34:1471–9. 10.1080/08912963.2022.2042807.

[59] Hernández Fernández M, Vrba ES. Macroevolutionary processes and biomic specialization: testing the resource-use hypothesis. Evol Ecol. 2005;19:199–219. 10.1007/s10682-004-8152-7.

[60] Hillebrand H. On the generality of the latitudinal diversity gradient. Am Nat. 2004;163:192–211. 10.1086/381004. 14970922 10.1086/381004

[61] Hillebrand H, Azovsky AI. Body size determines the strength of the latitudinal diversity gradient. Ecography. 2001;24:251–6. 10.1034/j.1600-0587.2001.240302.x.

[62] Jackson S. Gliding mammals of the world. Collingwood: Csiro Publishing; 2012.

[63] Janis CM, Ehrhardt D. Correlation of relative muzzle width and relative incisor width with dietary preference in ungulates. Zool J Linn Soc. 1988;92:267–84. 10.1111/j.1096-3642.1988.tb01513.x.

[64] Kaufman DM. Diversity of new world mammals: universality of the latitudinal gradients of species and bauplans. J Mammal. 1995;76:322–34. 10.2307/1382344.

[65] Klingenberg CP, Barluenga M, Meyer A. Shape analysis of symmetric structures-quantifying variation among individuals and asymmetry. Evolution. 2002;56:1909–20. 10.1111/j.0014-3820.2002.tb00117.x. 12449478 10.1111/j.0014-3820.2002.tb00117.x

[66] Koprowski JL, Goldstein EA, Bennett KR, Pereira Mendes C. Family Sciuridae (tree, flying and ground squirrels, chipmunks, marmots and prairie dogs). In: Wilson DE, Lacher Jr. TE, Mittermeier RA, editors. Handbook of the Mammals of the World: Vol. 6. Lagomorphs and Rodents I. Barcelona: Lynx Edicions; 2016. p. 648-837.

[67] Krause DW. Jaw movement, dental function, and diet in the Paleocene multituberculate *Ptilodus*. Paleobiology. 1982;8:265–81. 10.1017/S0094837300006989.

[68] Krishna M, Ray PC, Sarma K, Kumar A. Observations on particolored flying squirrel *Hylopetes alboniger* (Hodgson 1836) in Northeast India. Zoo’s Print. 2013;28:18–20.

[69] Lamanna C, Blonder B, Violle C, Kraft NJB, Sandel B, Šímová I, Donoghue JC II, Svenning J-C, McGill BJ, Boyle B, Buzzard V, Dolins S, Jørgensen PM, Marcuse-Kubitza A, Morueta-Holme N, Peet RK, Piel WH, Regetz J, Schildhauer M, Spencer N, Thiers B, Wiser SK, Enquist BJ. Functional trait space and the latitudinal diversity gradient. PNAS. 2014;111:13745–50. 10.1073/pnas.131772211. 25225365 10.1073/pnas.1317722111 PMC4183280

[70] Latorre DV. Padrões macroecólogicos de disparidade morfológica e distribuição de massa de mamíferos terrestres. São Paulo: University of São Paulo; 2015. 10.11606/D.41.2016.tde-26102015-111843

[71] Law CJ, Duran E, Hung N, Richards E, Santillan I, Mehta RS. Effects of diet on cranial morphology and biting ability in musteloid mammals. Evol Biol. 2018;31:1918–31. 10.1111/jeb.13385. 10.1111/jeb.13385 30270461

[72] Lee PF, Progulske DR, Lin YS. Ecological studies on two sympatric *Petaurista* species in Taiwan. Bull Inst Zool Acad Sin. 1986;25:113–23.

[73] Liu P-Y, Cheng A-C, Huang S-W, Lu H-P, Oshida T, Liu W, Yu H-T. Body-size scaling is related to gut microbial diversity, metabolism and dietary niche of arboreal folivorous flying squirrels. Sci Rep. 2020;10:7809. 10.1038/s41598-020-64801-y. 32385374 10.1038/s41598-020-64801-y PMC7210948

[74] Lu X, Ge D, Xia L, Huang C, Yang Q. Geometric morphometric study of the skull shape diversification in Sciuridae (Mammalia, Rodentia). Integr Zool. 2014;9:231–45. 10.1111/1749-4877.12035. 24952964 10.1111/1749-4877.12035

[75] MacArthur RH. Patterns of species diversity. Biol Rev Camb Philos Soc. 1965;40:510–33. 10.1111/j.1469-185X.1965.tb00815.x.

[76] Madden RH. Hypsodonty in mammals: Evolution, geomorphology and the role of Earth surface processes. Cambridge: Cambridge University Press; 2015.

[77] Maestri R, Patterson BD, Fornel R, Monteiro LR, De Freitas TRO. Diet, bite force and skull morphology in the generalist rodent morphotype. J Evol Biol. 2016;29:2191–204. 10.1111/jeb.12937. 27470674 10.1111/jeb.12937

[78] Manly GMO. Randomization, bootstrap and Monte Carlo methods in biology. London: Chapman & Hall; 1972. 10.1201/9781315273075

[79] Maser Z, Maser C, Trappe JM. Food habits of the northern flying squirrel (*Glaucomys sabrinus*) in Oregon. Can J Zool. 1985;63:1084–8. 10.1139/z85-162.

[80] McKenna MC. *Eupetaurus* and the living petauristine sciurids. Am Mus Novit. 1962;2104.

[81] Marugán-Lobón J, Blanco-Miranda D, Chamero Macho B, Martín-Abad H. On the importance of examining the relationship between shape data and biologically meaningful variables. an example studying allometry with geometric morphometrics. Span J Palaeont. 2013;28:139–48.

[82] Medrano González L. Morfología, función y dinámica de los organismos. In: Falconí Magaña M, editor. Esteva Peralta L. Biología matemática. Un enfoque desde los sistemas dinámicos. Ciudad de México: Universidad Nacional Autónoma de México; 2012. p. 19–39.

[83] Menéndez I, Gómez Cano AR, Cantalapiedra JL, Peláez-Campomanes P, Álvarez-Sierra MÁ, Hernández Fernández M. A multi-layered approach to the diversification of squirrels. Mamm Rev. 2021;51:66–81. 10.1111/mam.12215.

[84] Menéndez I, Swiderski DL, Gómez Cano AR, Hernández Fernández M, Álvarez-Sierra MÁ, Zelditch ML. Diet versatility and functional trade-offs shape tooth morphology in squirrels. Evolution. 2023;77:83–96. 10.1093/evolut/qpac019. 36689235 10.1093/evolut/qpac019

[85] Mittelbach GG, Schemske DW, Cornell HV, Allen AP, Brown JM, Bush MB, Harrison SP, Hurlbert AH, Knowlton N, Lessios HA, McCain CM, McCune AR, McDade LA, McPeek MA, Near TJ, Price TD, Ricklefs RE, Roy K, Sax DF, Schluter D, Sobel JM, Turelli M. Evolution and the latitudinal diversity gradient: speciation, extinction and biogeography. Ecol Lett. 2007;10:315–31. 10.1111/j.1461-0248.2007.01020.x. 17355570 10.1111/j.1461-0248.2007.01020.x

[86] Moreno Bofarull A, Royo AA, Hernández Fernández M, Ortiz-Jaureguizar E, Morales J. Influence of continental history on the ecological specialization and macroevolutionary processes in the mammalian assemblage of South America: Differences between small and large mammals. BMC Evol Biol. 2008;8:97. https://doi.org/10.1186/1471-2148-8-97 18366786 10.1186/1471-2148-8-97 PMC2330041

[87] Murray MG, Brown D. Niche separation of grazing ungulates in the Serengeti: an experimental test. J Anim Ecol. 1993;62:380–9. 10.2307/5369.

[88] Muul I. Behavioral and physiological influences on the distribution of the flying squirrel, *Glaucomys volans*. Misc publ - Mus Zool, Univ Mich. 1968;134:1–66.

[89] Muul I, Lim BL. Comparative morphology, food habits, and ecology of some Malaysian arboreal rodents. In: Montgomery GG, editor. The Ecology of Arboreal Folivores. Washington, D.C.: Smithsonian Institution Press; 1978. p. 361–8.

[90] Oliveira BF, Machac A, Costa GC, Brooks TM, Davidson AD, Rondinini C, Graham CH. Species and functional diversity accumulate differently in mammals. Glob Ecol Biogeogr. 2016;25:1119–30. 10.1111/geb.12471.

[91] Olsen A, Westneat M. StereoMorph: an R package for the collection of 3D landmarks and curves using a stereo camera set-up. Methods Ecol Evol. 2015;6:351–6. 10.1111/2041-210X.12326.

[92] Pagel M. Inferring the historical patterns of biological evolution. Nature. 1999;401:877–84. 10.1038/44766. 10553904 10.1038/44766

[93] Pečnerová P, Moravec JC, Martínková N. A skull might lie: modeling ancestral ranges and diet from genes and shape of tree squirrels. Syst Biol. 2015;64:1024–88. 10.1093/sysbio/syv054. 10.1093/sysbio/syv054 26254670

[94] Pérez-Barbería FJ, Gordon IJ. The functional relationship between feeding type and jaw and cranial morphology in ungulates. Oecologia. 1999;118:157–65. 10.1007/s004420050714. 28307690 10.1007/s004420050714

[95] Pianka ER. Latitudinal gradients in species diversity: a review of concepts. Am Nat. 1966;100:33–46. 10.1086/282398.

[96] Puttick M, Ingram T, Clarke M, Thomas G. MOTMOT: models of trait macroevolution on trees (an update). Methods Ecol Evol. 2020;11:464–71. 10.1111/2041-210X.13343.

[97] R Core Team. R: A language and environment for statistical computing. Vienna: R Foundation for Statistical Computing; 2021.

[98] Rahbek C. The elevational gradient of species richness: a uniform pattern? Ecography. 1995;18:200–5.

[99] Riesch R, Martin RA, Diamond SE, Jourdan J, Plath M, Brian LR. Thermal regime drives a latitudinal gradient in morphology and life history in a livebearing fish. Biol J Linn Soc. 2018;125:126–41. 10.1093/biolinnean/bly095.

[100] Rohlf FJ, Slice D. Extensions of the procrustes method for the optimal superimposition of landmarks. Syst Zool. 1990;39:40–59. 10.2307/2992207.

[101] Rohlf FJ, Marcus LF. A revolution in morphometrics. Trends Ecol Evol. 1993;8:129–32. 10.1016/0169-5347(93)90024-J. 21236128 10.1016/0169-5347(93)90024-J

[102] Roussel J, Barber CB, Habel K, Grasman R, Gramacy RB, Mozharovskyi P, Sterratt DC. Geometry: Mesh generation and Surface Tesselation. R package version 0.3-6; 2015.

[103] Safi K, Cianciaruso MV, Loyola RD, Brito D, Armour-Marshall K, Diniz-Filho JAF. Understanding global patterns of mammalian functional and phylogenetic diversity. Philos Trans R Soc B. 2011;366:2536–44. 10.1098/rstb.2011.0024. 10.1098/rstb.2011.0024 PMC3138614 21807734

[104] Samuels JX. Cranial morphology and dietary habits of rodents. Zool J Linn Soc. 2009;156:864–88. 10.1111/j.1096-3642.2009.00502.x.

[105] Sanamxay D, Douangboubpha B, Bumrungsri S, Xayavong S, Xayaphet V, Satasook C, Bates PJJ. Rediscovery of *Biswamoyopterus* (Mammalia: Rodentia: Sciuridae: Pteromyini) in Asia, with the description of a new species from Lao PDR. Zootaxa. 2013;3686:471–81. 10.11646/zootaxa.3686.4.5. 26473234 10.11646/zootaxa.3686.4.5

[106] Satoh K. Comparative functional morphology of mandibular forward movement during mastication of two murid rodents, *Apodemus speciosus* (Murinae) and *Clethrionomys rufocanus* (Arvicolinae). J Morphol. 1997;231:131–42. 8989873 10.1002/(SICI)1097-4687(199702)231:2<131::AID-JMOR2>3.0.CO;2-H

[107] Schmidt-Nielsen K. Energy metabolism, body size, and problems of scaling. Fed Proc. 1970;29:1524–32. 5459901

[108] Schmidt-Nielsen K. Scaling: Why is Animal Size so Important? Cambridge: Cambridge University Press; 1984.

[109] Shepherd U. A comparison of species diversity and morphological diversity across the North American latitudinal gradient. J Biogeogr. 1998;25:19–29. 10.1046/j.1365-2699.1998.251172.x.

[110] Small CG. The Statistical theory of shape. Berlin: Springer; 1996.

[111] Smith AT, Xie Y. A guide to the mammals of China. New Jersey: Princeton University Press; 2008.

[112] Solounias N, Moelleken SMC. Dietary adaptation of some extinct ruminants determined by premaxillary shape. J Mamm. 1993;74:1059–71. 10.2307/1382445.

[113] Spicer ME, Mellor H, Carson WP. Seeing beyond the trees: a comparison of tropical and temperate plan growth forms and their vertical distribution. Ecology. 2020;101:e02974. 10.1002/ecy.2974. 31944269 10.1002/ecy.2974

[114] Stevens GC. The latitudinal gradient in geographical range: how so many species coexist in the tropics? Am Nat. 1989;133:240–54. 10.1086/284913.

[115] Stevens RD, Willig MR, Strauss RE. Latitudinal gradients in the phenetic diversity of new World bat communities. Oikos. 2006;112:41–50. 10.1111/j.0030-1299.2006.13167.x.

[116] Suzuki K, Asari Y, Yanagawa H. Gliding locomotion of Siberian flying squirrels in low-canopy forests: the role of energy-inefficient short-distance glides. Acta Theriol. 2012;57:131–5. 10.1007/s13364-011-0060-y.

[117] Suzuki K, Yanagawa H. Gliding patterns of Siberian flying squirrels in relation to forest structure. iForest. 2019;12:114–7.

[118] Swiderski DL, Zelditch ML. Complex adaptive landscape for a “Simple” structure: the role of trade-offs in the evolutionary dynamics of mandibular shape in ground squirrels. Evolution. 2022;76:946–65. 10.1111/evo.14493. 35398910 10.1111/evo.14493 PMC9320833

[119] Symonds MRE, Blomberg SP. A primer on phylogenetic generalised least squares. In: Garamszegi LZ, editors. Modern Phylogenetic Comparative Methods and their Application in Evolutionary Biology. Berlin: Springer-Verlag; 2014. p. 105-130. 10.1007/978-3-662-43550-2_5

[120] Andreu MTC, Arlé CE, Minsky EMC, Laut L, da Rocha Fortes R, Lorini ML, Figueiredo MDSL. Thermoregulation or habitat selection? Environmental predictors of the body shape variation in sharks (Chondrichthyes: Selachimorpha). Oecologia Aust. 2018;22:179–90.

[121] Tennant JP, MacLeod N. Snout shape in extant ruminants. PLoS ONE. 2014;9:e112035. 10.1371/journal.pone.0112035. 25372878 10.1371/journal.pone.0112035 PMC4221202

[122] Thorington RW Jr, Heaney LR. Body proportions and gliding adaptations of flying squirrels (Petauristinae). J Mamm. 1981;62:101–14. 10.2307/1380481.

[123] Thorington RW Jr, Darrow K. Jaw muscles of old world squirrels. J Morphol. 1996;230:145–65. 8921609 10.1002/(SICI)1097-4687(199611)230:2<145::AID-JMOR3>3.0.CO;2-G

[124] Thorington RW Jr, Hoffmann RS. Family Sciuridae. In: Wilson DE, Reeder DM, editors. Mammal species of the world. Baltimore: The Johns Hopkins University Press; 2005. p. 754–818.

[125] Tong Y-J, Yang H-D, Shaw JJ, Yang XK, Bai M. The relationship between genus/species richness and morphological diversity among subfamilies of jewel beetles. Insects. 2021;12:24. 10.3390/insects12010024. 33401400 10.3390/insects12010024 PMC7830872

[126] Vázquez DP, Stevens RD. The latitudinal gradient in niche breadth: concepts and evidence. Am Nat. 2004;164:E1–19. 10.1086/421445. 15266376 10.1086/421445

[127] Wallace AR. Tropical nature, and other essays. London: Macmillan & Co; 1878.

[128] Walter H. Vegetation zones and climate. Stuttgart: Verlag Eugen Ulmer; 1970.

[129] Wang F. Preliminary study on the ecology of *Trogopterus xanthipes*. Acta Theriol Sin. 1985;5:103–10.

[130] Wickham H. ggplot2: Elegant graphics for data analysis. New York: Springer-Verlag; 2016.

[131] Williams SH, Kay RF. A comparative test of adaptive explanations for hypsodonty in ungulates and rodents. J Mamm Evol. 2001;8:207–29. 10.1023/A:1012231829141.

[132] Wilson DE, Lacher Jr TE, Mittermeier RA. Handbook of the Mammals of the World. vol. 6. Lagomorphs and Rodents I. Barcelona: Lynx Edicions; 2016.

[133] Wölfer J, Aschenbach T, Michel J, Nyakatura JA. Mechanics of arboreal locomotion in Swinhoe’s striped squirrels: a potential model for early Euarchontoglires. Front Ecol Evol. 2021;9:636039. 10.3389/fevo.2021.636039.

[134] Zahler P. The woolly flying squirrel and gliding: does size matter? Acta Theriol. 2001;46:429–35. 10.1007/BF03192449.

[135] Zahler P, Khan M. Evidence for dietary specialization on pine needles by the woolly flying squirrel (*Eupetaurus cinereus*). J Mamm. 2003;84:480–6. 10.1644/1545-1542(2003)084%3c0480:EFDSOP%3e2.0.CO;2.

[136] Zelditch ML, Swiderski DL, Sheets HD, Fink WL. Geometric morphometrics for biologists: a primer. New York: Elsevier Academic Press; 2004.

[137] Zelditch ML, Ye J, Mitchell JS, Swiderski DL. Rare ecomorphological convergence on a complex adaptive landscape: body size and diet mediate evolution of jaw shape in squirrels (Sciuridae). Evolution. 2017;71:633–49. 10.1111/evo.13168. 28075012 10.1111/evo.13168

